# Development and Characterization of Microsatellite Markers for the Euryhaline Polychaete *Laeonereis acuta* (Annelida: Nereididae) in the Southwestern Brazilian Coast

**DOI:** 10.1007/s10528-026-11364-8

**Published:** 2026-03-30

**Authors:** Pedro Caldas Esperon Carvalho, Paulo Cesar Paiva, Victor Corrêa Seixas

**Affiliations:** 1 Laboratório de Ecologia e Evolução Molecular, Departamento de Biologia Marinha, Instituto de Biologia, Universidade Federal Fluminense, Niterói, RJ Brasil; 2 Laboratório TAXOn, Departamento de Zoologia, Instituto de Biologia, Universidade Federal do Rio de Janeiro, Rio de Janeiro, RJ Brasil

**Keywords:** Population genetics, Annelida, Coastal lagoons, Molecular markers, Genetic diversity

## Abstract

*Laeonereis acuta* is a polychaete species typically found at high abundance in estuarine and coastal lagoon environments. Due to its association with polluted habitats, it is commonly used in ecotoxicological studies. Moreover, its occurrence in spatially discontinuous environments with high environmental variability makes it a suitable model for evolutionary studies of local adaptation, genetic landscape, and early stages of speciation. This study aimed to develop primers and characterize microsatellite markers for *L. acuta* sampled from three coastal lagoons in southwestern Brazil. A total of 10 loci were characterized based on the genotyping of 40 individuals. The number of alleles per locus ranged from 2 to 19. Evidence of null alleles was detected at five loci, although their frequency decreased when coastal lagoons were analyzed separately. When considering all individuals as a single population, five loci showed positive and significant *F*_*IS*_ values, and seven loci deviated from Hardy-Weinberg equilibrium. Maricá and Guarapina exhibited heterozygote excess at several loci, whereas Jaconé showed evidence of population genetic isolation. The 10 microsatellite loci were polymorphic and suitable for population genetic analysis in *L. acuta*, although these patterns may not necessarily be representative of other geographic regions. These markers may contribute to ecotoxicological studies by clarifying whether physiological responses to pollutants are associated with genetic differentiation among populations. Furthermore, they provide valuable tools for investigating genetic structure and connectivity in discontinuous environments.

**Graphical Abstract:**

**Figure:**
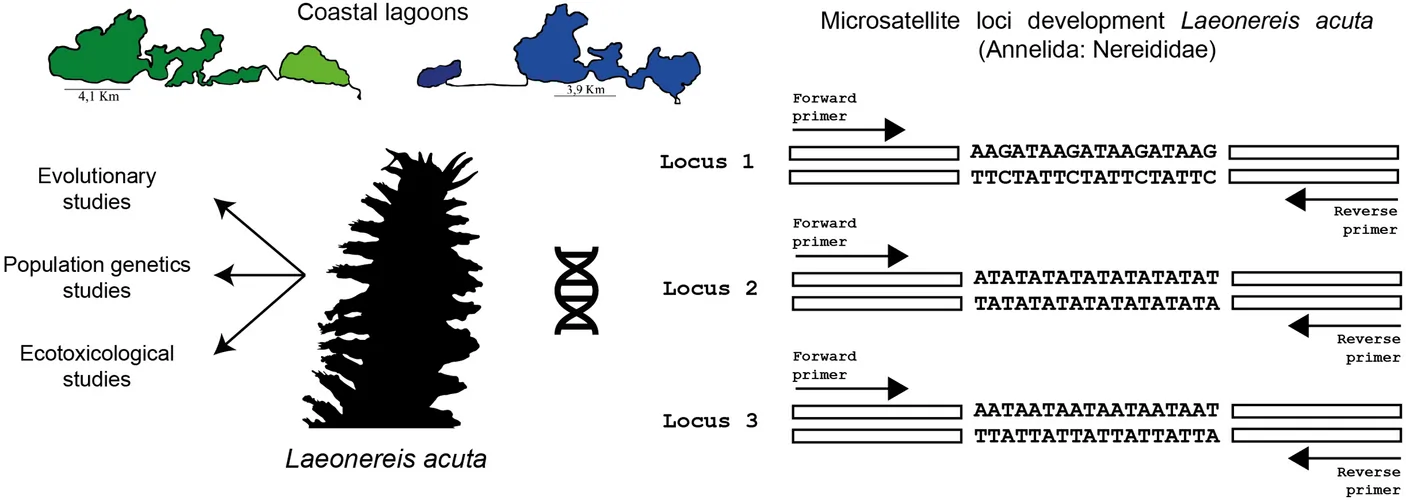

**Supplementary Information:**

The online version contains supplementary material available at https://doi.org/10.1007/s10528-026-11364-8.

## Introduction

The geographic distribution of species is determined by ecological processes that include abiotic factors, such as historical (i.e., climate changes) and contemporary (i.e., ecological gradients), as well as biotic factors, including life-history traits, ecological interactions, and requirements (Brown et al. 1996). Concurrently, the ability of individuals within a population to survive abiotic and biotic interactions depends on their genetic composition, which is shaped by the evolutionary forces (Saccheri and Hanski 2006). While natural selection and genetic drift tend to decrease intra-population genetic diversity and increase inter-population genetic differentiation, mutation and gene flow increase diversity, and the latter can also homogenize population gene pool (Slatkin 1987). Gene flow, therefore, promotes the spread of evolutionary novelties, while migration allows the recolonization of locally extinct populations, offsets negative recruitment rates, and influences geographic range distribution (Lowe and Allendorf 2010).

In this context, the use of highly polymorphic molecular markers, such as microsatellites, is essential for understanding the contemporary genetic landscape in which populations of different species are embedded (Epps and Keyghobadi 2015). Located in non-coding nuclear genomic regions, microsatellites generally evolve neutrally (Ellegren 2004) and have been widely used in studies of kinship, parentage analysis, and genetic diversity (Wenne 2023). Although reduced by high-throughput sequencing (HTS) technologies, microsatellite development still represents an initial investment that becomes lower once primers are validated. Single-nucleotide polymorphisms (SNPs) represent an alternative that can be applied on a genome-wide scale; however, their use in population studies often remains costly (Flanagan and Jones 2019). Thus, the development of microsatellite primers represents a cost-effective approach for population genetic studies.

Studies on genetic connectivity can be useful for determining source/sink populations and the degree of population confinement and may therefore provide relevant information for the development of conservation strategies (Alves et al. 2023). Furthermore, information on genetic connectivity enables investigations into allele sharing, the emergence of genetic breaks, and the speciation process (Monti et al. 2018). Within an evolutionary framework, discontinuous environments such as estuaries and coastal lagoons provide valuable settings for examining local adaptations, population genetic structure, and the early stages of lineage diversification (Dool et al. 2022).

Among the taxa inhabiting these environments, species of the genus *Laeonereis* (Annelida: Nereididae) stand out due to their richness, abundance, and dominance in estuarine and coastal lagoon environments (Martin and Bastida 2006; Mendes and Soares-Gomes 2011). The genus is widely distributed along the western Atlantic coast from Maryland (USA; 38°N) to Puerto Madryn (Argentina; 42°S) and tolerates high salinity fluctuations, occurring from low salinity to hypersaline systems (Seixas et al. 2018; Silva et al. 2025 preprint). Although *L. culveri* was once considered the only species in the genus (Pettibone 1971), at least seven species are now recognized (Conde-Vela 2021; Sampieri et al. 2021).

Due to the capability to absorb heavy metals and organic pollutants from sediment, species such as *L. culveri* and *L. acuta* are frequently used in ecotoxicological studies that assess the effects of different pollutant types (e.g., organic pollution, metal, and pesticides) on organism physiology (Sun and Zhou, 2007; Aguiar et al. 2023; Müller et al. 2025). Because physiological and ecotoxicological responses may vary among populations depending on local adaptation and life history traits (e.g., reproductive strategies, spawning period, egg size) – which generally reflect population genetic variation – the study of population genetic structure and connectivity becomes particularly important. Such studies can reveal the sharing of relevant alleles among populations. In this context, microsatellites have proven effective in revealing population genetic structure in estuarine and coastal lagoon species, such as the horseshoe crab *Limulus polyphemus* (Zamora-Bustillos et al. 2023; Noreña-iva, et al. 2023) and the fishes *Mugil curema* (Pacheco-Almanzar et al. 2017), *Atherina boyeri* (Milana et al. 2012), and *Pomatoschistus marmoratus* (González-Wangüemert and Vergara-Chen 2014).

The present study aimed to characterize microsatellite loci for the polychaete *Laeonereis acuta* (Treadwell, 1923), an abundant inhabitant of coastal lagoons. These environments are spatially discontinuous and physically regulated, characteristics that tend to promote population fragmentation and local adaptation. Consequently, coastal lagoon species are suitable models for investigating fine-scale population genetic divergence and the early stages of speciation. In such systems, assessment of genetic structure, gene flow, and kinship requires highly polymorphic molecular markers. The microsatellite loci characterized here provide essential tools for population genetics, evolutionary, and ecotoxicological studies.

## Materials and Methods

For primer development, DNA was extracted from a single *L. acuta* specimen collected in Maricá Lagoon (22°57’16” S; 42°53’4” W) using the Blood and Tissue kit (Qiagen), following the manufacturer’s protocol for alcohol-fixed tissue. High-throughput sequencing was performed on a GS FLX 454 (Roche) platform to identify microsatellite regions. A total of 135,082 sequences were obtained, ranging from 56 to 1,780 bp (median = 1,052 bp). After filtering for reads with an average quality score > 25, QDD 3.1 (Meglécz et al. 2014) was used to (a) identify reads containing microsatellites, (b) select unique reads, and (c) design primers.

In total, 399 microsatellite groups and more than 11,000 primer options were identified. From these, only pure and uninterrupted microsatellites with at least eight repeats, the lowest alignment score within the group, and a product size of 150–500 bp were selected. After filtering, 48 primer sets remained, of which 15 were selected for testing: six dinucleotide, four trinucleotide, two tetranucleotide, and three pentanucleotide loci.

**Fig. 1:**
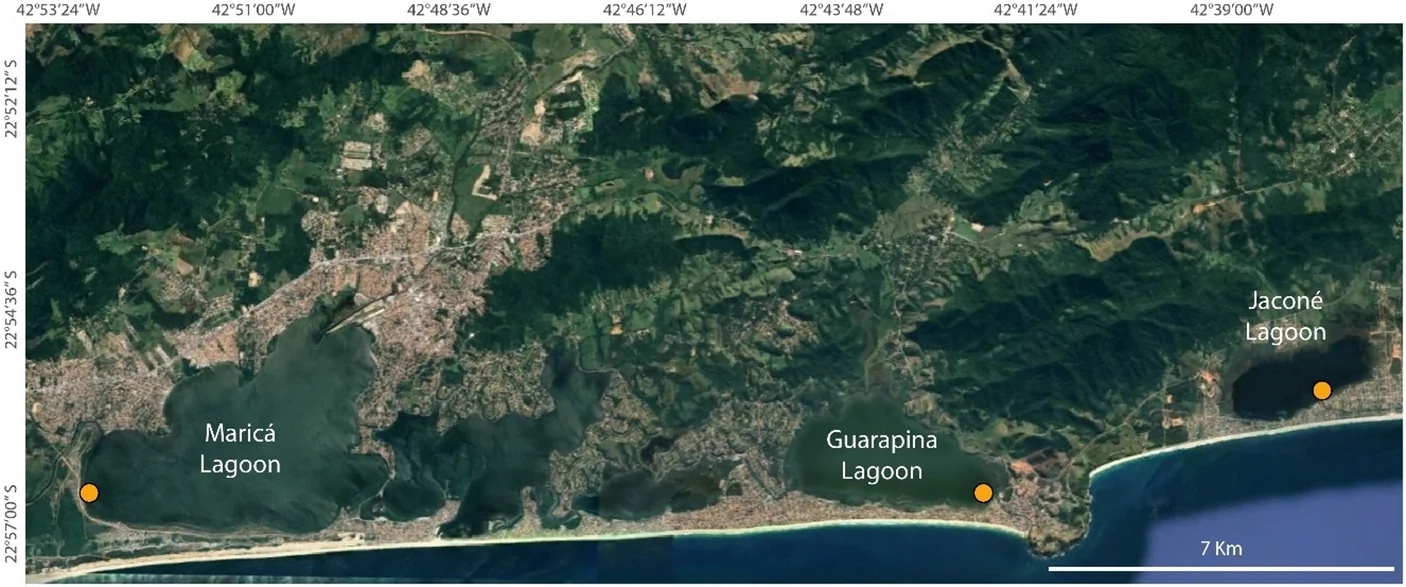
Map of the sampling sites for *Laeonereis acuta* in three coastal lagoons along the southwestern Brazilian coast, Rio de Janeiro State. Maricá Lagoon (22°57’7.77” S 42°53’5.31” W); Guarapina Lagoon (22°57’5.92” S 42°42’7.85” W) and Jaconé Lagoon (22°55’54.69” S 42°38’6.10” W)

To test primer effectiveness, individuals from three coastal lagoons in southeastern Brazil (Rio de Janeiro) were sampled in 2023: Maricá Lagoon (n = 12; 22°57’7.77” S 42°53’5.31” W), Guarapina Lagoon (*n* = 12; 22°57’5.92” S 42°42’7.85” W) and Jaconé Lagoon (*n* = 16: 22°55’54.69” S 42°38’6.10” W) (Fig. 1). Sampling in each coastal lagoon was carried out randomly within an area of approximately 10 m^2^ using a shovel and sieve. Specimens were preserved in 95% ethanol in the field and subsequently stored at −20 °C. Individuals were morphologically identified following the *Laeonereis* revision of Conde-Vela (2021). In addition, specimens from these coastal lagoons were previously molecularly identified as belonging to the same molecular operational taxonomic unit (Seixas et al. 2018; Sampieri et al. 2021). DNA extraction was performed using the Puregene Tissue kit (Qiagen), following the manufacturer’s protocol for alcohol-fixed tissue. Specimens of different sizes were selected to minimize the probability of sampling individuals from the same cohort.

Based on the estimated melting temperature of forward and reverse primers (57–60 °C), primer optimization began with an annealing temperature of 58 °C. Although some primer sets amplified at 58 °C, temperature gradient tests indicated that annealing at 52 °C was optimal for all 10 primer sets (five of the 15 primer sets failed to amplify at any tested temperature). To reduce genotyping costs, the Schuelke (2000) approach was applied, using forward primers with an M13 tail and fluorochrome-labeled M13 primers (6-FAM, VIC, PET, and NED). PCRs were performed in a 10 µL final volume reaction containing: 1X buffer, 2.5 mM MgCl2, 1 mg/mL BSA, 0.2 mM of each dNTP, 0.2 µM of forward primer with M13 tail, 0.8 µM of reverse primer, 0.4 µM of fluorochrome-labeled M13 primers, and 0.025U of DNA polymerase (GoTaq G2 Hot Start, Promega). Cycling conditions were: initial denaturation cycle at 95 °C for 2 min; five cycles of 95 °C for 30 s, 52–58 °C for 30 s, and 72 °C for 45 s; followed by 35 cycles of 92 °C for 30 s, 52 °C for 30 s, and 72 °C for 55 s; with a final extension at 72 °C for 5 min. Negative controls were used in all PCRs, and amplification success was verified on 2% agarose gel using a 100 bp DNA ladder (Thermo Scientific). Genotyping was performed on a SeqStudio sequencer (Thermo Scientific) using the GeneScan 600 LIZ size standard. Microsatellite sequences were deposited in the DNA Data Bank of Japan (DDBJ) (*Lae_02*-LC896134, *Lae_03* - LC896135, *Lae_04*-LC896136, *Lae_05* - LC896137, *Lae_06* - LC896138, *Lae_08* - LC896139, *Lae_10* - LC896140, *Lae_11* - LC896141, Lae_14 - LC896142, *Lae_15* - LC896143).

Allele sizes were determined using the OSIRIS 2.16 program (www.ncbi.nlm.nih.gov/osiris/). The presence of null alleles, scoring errors due to stuttering, and large allele dropout was assessed using MICRO-CHEKER 2.2.0.3 (Oosterhout et al. 2004). Linkage disequilibrium was tested in Genepop 4.7 (Rousset 2008). The number of alleles and rarefied allelic richness were estimated using the R package *hierstat* (Goudet 2005). Observed and expected heterozygosity, inbreeding coefficient (*F*_*IS*_), deviations from the Hardy-Weinberg equilibrium (HWE), and pairwise *F*_*ST*_ were calculated using Arlequin 3.5 (Excoffier et al. 2005). Pairwise *F*_*ST*_ was calculated allowing up to 20% missing data. Significant levels were adjusted using the false discovery rate (FDR) method (Benjamini and Hochberg 1995), as implemented in the R package *stats*. Because specimens within each coastal lagoon were sampled at close spatial distances, kinship relationships among individuals were inferred using a maximum-likelihood approach implemented in ML-Relate (Kalinowski et al. 2006), which classified pairwise relationships as unrelated, half-siblings, full-siblings, and parent-offspring.

## Results and Discussion

Across the 40 genotyped individuals, 74 alleles were detected in 10 microsatellite loci. The overall percentage of missing data was 12.5%, with locus *Lae_10* being the least represented (*n* = 28) and loci *Lae_06* and *Lae_11* being the most represented (*n* = 40) (Table 1). No loci showed evidence of large-allele dropout or scoring errors due to stuttering, except for *Lae_5* (Table 1). Potential null alleles were detected at five loci (*Lae_02*, *Lae_03*, *Lae_05*, *Lae_10*, and *Lae_15*) (Table 1), although their frequency decreased when loci were analyzed separately for each coastal lagoon (see below). No pairs of loci showed significant linkage disequilibrium (Table S1 – Supplementary Material). Comparisons involving locus *Lae_06* were not computed due to the low allelic variability.

**Table 1:** Primer sequences and locus parameters for 10 microsatellite markers described for *Laeonereis acuta*

Loci	Primer sequence (5’−3’)	Motif	Size range	*N*	Na	Ho	He	F_IS_	HWE	LAD	SE	Null
Lae_02	F: ACAAACTGCAGAGTCACAAACG	AAGAT	416–471	37	10	0.676	0.824	**0.194**	0.228	no	no	yes
	R: AGCTGCAGATTTCCTCAGCG											
Lae_03	F: CAACTTCCTGCATTCCACGC	AAT	208–238	33	7	0.424	0.662	**0.372**	**0.002**	no	no	yes
	R: ATGAATGTGAACTGTATGAACGTT											
Lae_04	F: TGGCTAATGTTGAAACGGCTC	AATAG	345–420	33	10	0.697	0.818	0.163	**0.023**	no	no	no
	R: ACCGAGGATTATCCATATGCTCT											
Lae_05	F: AACACTCACTCGTAAGGCCC	AG	295–297	37	2	0.297	0.463	**0.370**	**0.049**	no	yes	yes
	R: AACGCGATGGGAAATGCAGA											
Lae_06	F: GTCTTACCGCTCTGAGTGCA	AG	340–346	40	2	0.600	0.420	−0.418	**0.020**	no	no	no
	R: GGACGGGTAATGTCAGGGTG											
Lae_08	F: TCTGATGGAAATCACCGGCT	AGAT	330–426	37	19	0.838	0.887	0.069	**0.008**	no	no	no
	R: GCACAAACCCACAGTTTCGG											
Lae_10	F: GCCTTGGTCATTGCGTTACA	AT	453–465	28	3	0.143	0.465	**0.702**	**< 0.001**	no	no	yes
	R: GTTCGTTAGAAAGCGTCGGC											
Lae_11	F: TGTTCAGTGGCTGGTGATGT	AT	232–242	40	3	0.400	0.417	0.052	0.493	no	no	no
	R: GCTGTGTGCTGCAAATGAAA											
Lae_14	F: TCATGTTATGCACTGTCTGGCT	ATC	457–484	32	6	0.625	0.604	−0.020	0.648	no	no	no
	R: CATTCTTGGCAGCAACGTCA											
Lae_15	F: GCTGTTCTTGTCTTCAACTGGA	ATC	406–466	32	12	0.656	0.846	**0.239**	**0.023**	no	no	yes
	R: GGGTCTTTATTGTTGCGTAATCT											

Bold values indicate significant values (all p-values were adjusted using the false discovery rate (FDR) method). *N* number of samples, *Na* number of alleles, *Ar* allelic richness, *Ho* observed heterozygosity, *He* expected heterozygosity, *HWE* Hardy-Weinberg equilibrium, *LAD* large allele dropout, *SE* scoring error due to stuttering, Null – null alleles

The number of alleles per locus ranged from two (*Lae_05* and *Lae_06*) to 19 (*Lae_08*) (Table 1), with a mean of 7.4 alleles per locus (SD = 5.46). Dinucleotide loci showed the lowest number of alleles (mean = 2.5; SD = 0.58), followed by trinucleotide loci (mean = 8.33; SD = 3.2). Tetra- and pentanucleotide loci exhibited 10 or more alleles each. These values are within the range reported for other polychaetes, such as the nereidid *Platynereis dumerilli* (Popa et al. 2014) and the siboglinid *Seepiophila jonesi* (Huang et al. 2016), as well as other invertebrates, such as the sponge *Mycale cecilia* (Hernández-Lozano et al. 2023) and the bivalve *Atrina vexillum* (Guttierez et al. 2025).

Observed heterozygosity was lower than expected for all loci except *Lae_06* and *Lae_14*. Seven loci deviated significantly from HWE (*Lae_03*, *Lae_04*, *Lae_05*, *Lae_06*, *Lae_08*, *Lae_10*, *Lae_15*), and five showed significant and positive *F*_*IS*_ values (*Lae_02*, *Lae_03*, *Lae_05*, *Lae_10*, *Lae_15*) (Table 1). The overall *F*_*IS*_ across all samples was 0.178 (*p* < 0.0001). Deviations from HWE and positive *F*_*IS*_ may result from non-random mating, such as inbreeding, or from the presence of genetically structured populations (Keller and Waller 2002). In addition, a small sample size can affect HWE tests by reducing statistical power and increasing sampling variance (Waples 2015).

Pairwise *F*_*ST*_ values indicate no genetic differentiation between Marica and Guarapina (MAR×GUA - *F*_*ST*_ = 0.0016; *p* = 0.16), whereas Jaconé differed significantly from both (JAC×MAR - *F*_*ST*_ = 0.1718, *p* < 0.0001; JAC×GUA - *F*_*ST*_ = 0.1933, *p* < 0.0001). Considering each coastal lagoon separately, the total number of alleles detected was 46 in Maricá, 38 in Guarapina, and 47 in Jaconé. No loci showed evidence of large allele dropout or scoring errors due to stuttering, and null alleles were detected only at loci *Lae_03*, *Lae_10*, and *Lae_15* in Jaconé and at *Lae_10* in Guarapina (Table 2). Only a few loci per coastal lagoon deviated significantly from HWE (Table 2). Overall, the number of shared alleles exceeded the number of private alleles (Table S2 – Supplementary Material). Maricá Lagoon exhibited more private alleles at *Lae_02*, *Lae_08*, *Lae_10*, and *Lae_11*, whereas Jaconé had more private alleles at *Lae_03*, *Lae_04*, *Lae_14*, and *Lae_15*. Alleles at *Lae_05* and *Lae_06* were shared among all coastal lagoons. Although Jaconé Lagoon exhibited more private alleles, the number of shared alleles was generally equivalent.

**Table 2:** Locus parameters for 10 microsatellite markers of *Laeonereis acuta*, considering each coastal lagoon

		Locus									
	Site	Lae_02	Lae_03	Lae_04	Lae_05	Lae_06	Lae_08	Lae_10	Lae_11	Lae_14	Lae_15
N	Maricá	11	9	10	11	12	11	8	12	12	9
	Guarapina	12	12	8	12	12	10	10	12	4	8
	Jaconé	14	12	15	14	16	16	10	16	16	15
Na	Maricá	8	4	5	2	2	10	3	3	3	6
	Guarapina	8	3	5	2	2	5	2	2	3	6
	Jaconé	5	6	8	2	1	8	2	2	5	8
Ar	Maricá	4.99	3.15	4.08	1.76	2.00	5.76	2.67	2.30	2.33	4.38
	Guarapina	4.52	2.39	3.50	1.83	2.00	3.19	1.90	2.00	3.00	4.17
	Jaconé	3.76	4.03	4.77	1.96	1.00	4.46	2.00	1.59	3.22	4.90
Ho	Maricá	0.91	0.67	0.80	0.27	1.00	0.82	0.38	0.58	0.75	0.78
	Guarapina	0.58	0.33	0.50	0.33	1.00	0.90	0.00	0.67	0.75	0.62
	Jaconé	0.57	0.33	0.73	0.29	.	0.81	0.10	0.06	0.50	0.60
He	Maricá	0.81	0.64	0.74	0.24	0.50	0.86	0.48	0.47	0.53	0.75
	Guarapina	0.75	0.40	0.66	0.28	0.50	0.62	0.32	0.50	0.59	0.69
	Jaconé	0.72	0.74	0.80	0.41	.	0.79	0.49	0.17	0.57	0.82
F_IS_	Maricá	−0.075	0.010	−0.021	−0.111	−1.000	0.091	0.276	−0.203	−0.375	0.026
	Guarapina	0.267	0.214	0.300	−0.158	−1.000	−0.409	1.000	−0.294	−0.125	0.157
	Jaconé	0.244	**0.579**	0.120	0.333	.	−0.003	0.816	0.651	0.149	0.300
HWE	Maricá	1.000	0.319	0.457	1.000	**0.019**	0.457	0.457	0.457	0.457	1.000
	Guarapina	0.050	0.516	0.516	1.000	**0.019**	0.516	**0.046**	0.704	1.000	0.516
	Jaconé	0.519	**0.007**	0.165	0.368	.	0.519	0.074	0.165	0.150	0.156
LAD	Maricá	no	no	no	no	no	no	no	no	no	no
	Guarapina	no	no	no	no	no	no	no	no	no	no
	Jaconé	no	no	no	no	no	no	no	no	no	no
SE	Maricá	no	no	no	no	no	no	no	no	no	no
	Guarapina	no	no	no	no	no	no	no	no	no	no
	Jaconé	no	no	no	no	no	no	no	no	no	no
Null	Maricá	no	no	no	no	no	no	no	no	no	no
	Guarapina	no	no	no	no	no	no	yes	no	no	no
	Jaconé	no	yes	no	no	no	no	yes	no	no	yes

Bold values indicate significant values (all p-values were adjusted using the false discovery rate (FDR) method)*. N* number of samples, *Na* number of alleles, *Ar* allelic richness, *Ho* observed heterozygosity, *He* expected heterozygosity, *HWE* Hardy-Weinberg equilibrium, *LAD* large allele dropout, *SE,* scoring error due to stuttering, Null – null alleles

In Maricá and Guarapina, six and five loci, respectively, showed heterozygote excess and negative (but non-significant) *F*_*IS*_ values (Table 2). The overall *F*_*IS*_ in Marica and Guarapina were − 0.105 (*p* = 0.954) and − 0.009 (*p* = 0.547), respectively. The hHeterozygote excess may reflect gene flow or a recent population bottleneck (Keller and Waller 2002). Maricá and Guarapina lagoons belong to the same coastal lagoon system and are connected by channels (Knoppers et al. 1991), which may facilitate gene flow. In contrast, loci from Jaconé suggested higher levels of inbreeding (Table 2; overall *F*_*IS*_ = 0.309, *p* < 0.001). Water renewal in Jaconé Lagoon depends on a narrow artificial channel connecting it to another choked lagoon (i.e., Saquarema Lagoon), resulting in a high degree of confinement (Mendes and Soares-Gomes 2011). Thus, elevated inbreeding levels are consistent with the geomorphological characteristics of Jaconé Lagoon.

The number of alleles, including all loci, was 46 in Marica, 38 in Guarapina, and 47 in Jaconé. Rarefied allelic richness for each locus was higher in Jaconé for five loci and higher in Maricá for four loci (Table 2). Both Maricá and Jaconé coastal lagoons are highly confined systems with limited seawater influence (Knoppers et al. 1991). Genetic diversity (allelic richness and heterozygosity) was expected to be lower in confined systems, whereas Guarapina Lagoon, as a less confined system, was expected to support higher diversity due to greater immigration potential. This pattern parallels species richness gradients across coastal lagoons with varying degrees of confinement (Barnes et al. 2013).

The lower genetic diversity observed in Guarapina may be related to kinship structure (Table S3 – Supplementary Material). Intrapopulation kinship analyses revealed a predominance of unrelated pairs (U), with 63.6% in Maricá, 42.4% in Guarapina, and 63.6% in Jaconé. The proportion of half-sibling (HS), full-sibling (FS), and parental-offspring (PO) was higher in Guarapina (57.6%; HS = 24.2%; FS = 27.3%; PO = 6.1%) than in Jaconé (38,2%; HS = 29.4%; FS = 7.4%; PO = 1.5%) and Maricá (36,4%; HS = 19.7%; FS = 10.6%; PO = 6.1%) (Table S3). In interpopulation comparisons, most pairs were classified as unrelated: 72.9% (Maricá × Guarapina), 98.4% (Maricá × Jaconé), and 97.9% (Guarapina × Jaconé) (Table S3).

Considering that *L. culveri* incubates its eggs in tubes and exhibits direct development (Mazurkiewicz 1975), *L. acuta* is also expected to have limited dispersal potential and high kinship at a small spatial scale. Consequently, seawater inflow may not necessarily facilitate immigration in *L. acuta* populations, and a positive correlation between coastal lagoon openness and genetic diversity may not be expected. These results ​​contrast with patterns inferred from mitochondrial COI sequences in the same geographical region (Seixas et al. 2018) and in other estuarine or coastal lagoon taxa (Dupont et al. 2009). This discrepancy likely reflects differences in temporal resolution: microsatellites capture contemporary fine-scale genetic structure and connectivity, whereas mitochondrial markers integrate processes over longer evolutionary timescales.

## Conclusions

This study reports the characterization of 10 microsatellite loci for the polychaete *L. acuta* and provides primer sequences for amplification. Most loci were polymorphic and proved suitable for detecting fine-scale population genetic subdivision, highlighting their utility for ecological and evolutionary studies. These markers may also support ecotoxicological research by enabling assessments of whether genetically isolated populations exhibit distinct physiological responses to environmental stressors. Moreover, applying these loci to estuarine and coastal lagoon regions beyond southeastern Brazil may help elucidate patterns and levels of genetic connectivity among populations inhabiting spatially discontinuous environments.

## Supplementary Information

Below is the link to the electronic supplementary material.

Supplementary Material 1.

## Data Availability

No datasets were generated or analysed during the current study.
