# Supplementary material for: Development and Characterization of Microsatellite Markers for the Euryhaline Polychaete Laeonereis acuta (Annelida: Nereididae) in the Southwestern Brazilian Coast
Source: Biochem Genet. 2026 Mar 30;64(5):7808–20. doi: 10.1007/s10528-026-11364-8 (PMC13547163; doi:10.1007/s10528-026-11364-8)
Supplement: Supplementary file 1 [file 10528_2026_11364_MOESM1_ESM.docx]

**Supplementary Material**

**Development and characterization of microsatellite markers for the euryhaline polychaeta *Laeonereis acuta* (Annelida: Nereididae) in the southwestern Brazilian coast**

Pedro Caldas Esperon Carvalho^1^, Paulo Cesar Paiva^2^, Victor Corrêa Seixas^1,*^

^1^Laboratório de Ecologia e Evolução Molecular, Departamento de Biologia Marinha, Instituto de Biologia, Universidade Federal Fluminense, Niterói-RJ, Brasil

^2^ Laboratório TAXOn, Departamento de Zoologia, Instituto de Biologia, Universidade Federal do Rio de Janeiro, Rio de Janeiro, RJ - Brasil

*Corresponding author: vcseixas@id.uff.br

ORCID Numbers:

Paulo Cesar Paiva – ORCID: 0000-0003-1061-6549

Victor Corrêa Seixas – ORCID: 0000-0002-2067-9490

**Table S1.** Linkage disequilibrium test between pairs of loci based on Fisher’s method. The locus Lae_06 (loc_06) has no variation.

| Locus pair | | Chi-square | df | p-value |
| --- | --- | --- | --- | --- |
| loc_03 | loc_04 | 0.0000 | 6 | 1.0000 |
| loc_03 | loc_08 | 5.9547 | 6 | 0.4283 |
| loc_04 | loc_08 | 2.9880 | 6 | 0.8103 |
| loc_03 | loc_15 | 0.0000 | 6 | 1.0000 |
| loc_04 | loc_15 | 9.8846 | 6 | 0.1296 |
| loc_08 | loc_15 | 0.0000 | 4 | 1.0000 |
| loc_03 | loc_14 | 3.3397 | 6 | 0.7652 |
| loc_04 | loc_14 | 0.7924 | 4 | 0.9395 |
| loc_08 | loc_14 | 2.5207 | 4 | 0.6409 |
| loc_15 | loc_14 | 0.0000 | 4 | 1.0000 |
| loc_03 | loc_05 | 8.2487 | 6 | 0.2204 |
| loc_04 | loc_05 | 4.6658 | 6 | 0.5873 |
| loc_08 | loc_05 | 0.9760 | 4 | 0.9134 |
| loc_15 | loc_05 | 3.6628 | 6 | 0.7222 |
| loc_14 | loc_05 | 4.2319 | 6 | 0.6453 |
| loc_03 | loc_10 | 4.1806 | 6 | 0.6523 |
| loc_04 | loc_10 | 1.3621 | 6 | 0.9681 |
| loc_08 | loc_10 | 1.0634 | 4 | 0.9000 |
| loc_15 | loc_10 | 5.6305 | 6 | 0.4658 |
| loc_14 | loc_10 | 0.9253 | 4 | 0.9209 |
| loc_05 | loc_10 | 1.5247 | 6 | 0.9578 |
| loc_03 | loc_02 | 3.8334 | 4 | 0.4290 |
| loc_04 | loc_02 | 3.1127 | 4 | 0.5391 |
| loc_08 | loc_02 | 3.5878 | 4 | 0.4647 |
| loc_15 | loc_02 | 0.0000 | 2 | 1.0000 |
| loc_14 | loc_02 | 1.5502 | 6 | 0.9561 |
| loc_05 | loc_02 | 4.1396 | 6 | 0.6578 |
| loc_10 | loc_02 | 3.2324 | 4 | 0.5197 |
| loc_03 | loc_11 | 0.9936 | 6 | 0.9859 |
| loc_04 | loc_11 | 7.4803 | 6 | 0.2787 |
| loc_08 | loc_11 | 2.6525 | 6 | 0.8510 |
| loc_15 | loc_11 | 2.4428 | 6 | 0.8748 |
| loc_14 | loc_11 | 2.2225 | 6 | 0.8981 |
| loc_05 | loc_11 | 0.5204 | 6 | 0.9976 |
| loc_10 | loc_11 | 4.4147 | 6 | 0.6207 |
| loc_02 | loc_11 | 7.4153 | 6 | 0.2841 |
| loc_03 | loc_06 | Not possible. | | |
| loc_04 | loc_06 | Not possible. | | |
| loc_08 | loc_06 | Not possible. | | |
| loc_15 | loc_06 | Not possible. | | |
| loc_14 | loc_06 | Not possible. | | |
| loc_05 | loc_06 | Not possible. | | |
| loc_10 | loc_06 | Not possible. | | |
| loc_02 | loc_06 | Not possible. | | |
| loc_11 | loc_06 | Not possible. | | |

**Table S2.** Allele frequency per locus and sampling site. Mar – Maricá Lagoon; Gua – Guarapina Lagoon; Jac – Jaconé Lagoon.

| **Locus** | Allele frequency | | | | | | | | | | | | | | | | | | |  | Allele | | | |
| --- | --- | --- | --- | --- | --- | --- | --- | --- | --- | --- | --- | --- | --- | --- | --- | --- | --- | --- | --- | --- | --- | --- | --- | --- |
| **Lae_02** | 416 | 421 | 426 | 441 | 446 | 451 | 456 | 461 | 466 | 471 |  |  |  |  |  |  |  |  |  |  | Total | Private | Shared |  |
| Mar | 0.14 | 0 | 0.05 | 0.05 | 0.27 | 0.27 | 0.09 | 0.05 | 0.09 | 0 |  |  |  |  |  |  |  |  |  |  | 8 | 2 | 6 |  |
| Gua | 0.13 | 0.04 | 0 | 0 | 0.04 | 0.21 | 0.04 | 0.08 | 0.42 | 0.04 |  |  |  |  |  |  |  |  |  |  | 8 | 1 | 7 |  |
| Jac | 0 | 0 | 0 | 0 | 0 | 0.14 | 0.14 | 0.39 | 0.29 | 0.04 |  |  |  |  |  |  |  |  |  |  | 5 | 0 | 5 |  |
| **Lae_03** | 208 | 211 | 223 | 226 | 229 | 232 | 238 |  |  |  |  |  |  |  |  |  |  |  |  |  | Total | Private | Shared |  |
| Mar | 0.44 | 0 | 0.11 | 0.06 | 0 | 0.39 | 0 |  |  |  |  |  |  |  |  |  |  |  |  |  | 4 | 1 | 3 |  |
| Gua | 0.08 | 0 | 0 | 0.17 | 0 | 0.75 | 0 |  |  |  |  |  |  |  |  |  |  |  |  |  | 3 | 0 | 3 |  |
| Jac | 0.33 | 0.08 | 0 | 0.04 | 0.04 | 0.33 | 0.17 |  |  |  |  |  |  |  |  |  |  |  |  |  | 6 | 3 | 3 |  |
| **Lae_04** | 345 | 365 | 370 | 375 | 380 | 385 | 390 | 395 | 400 | 420 |  |  |  |  |  |  |  |  |  |  | Total | Private | Shared |  |
| Mar | 0 | 0 | 0 | 0.1 | 0.35 | 0.3 | 0.1 | 0.15 | 0 | 0 |  |  |  |  |  |  |  |  |  |  | 5 | 1 | 4 |  |
| Gua | 0 | 0 | 0.06 | 0 | 0.44 | 0.38 | 0.06 | 0.06 | 0 | 0 |  |  |  |  |  |  |  |  |  |  | 5 | 1 | 4 |  |
| Jac | 0.27 | 0.07 | 0 | 0 | 0.1 | 0.1 | 0.1 | 0.3 | 0.03 | 0.03 |  |  |  |  |  |  |  |  |  |  | 8 | 4 | 4 |  |
| **Lae_05** | 295 | 297 |  |  |  |  |  |  |  |  |  |  |  |  |  |  |  |  |  |  | Total | Private | Shared |  |
| Mar | 0.86 | 0.14 |  |  |  |  |  |  |  |  |  |  |  |  |  |  |  |  |  |  | 2 | 0 | 2 |  |
| Gua | 0.83 | 0.17 |  |  |  |  |  |  |  |  |  |  |  |  |  |  |  |  |  |  | 2 | 0 | 2 |  |
| Jac | 0.29 | 0.71 |  |  |  |  |  |  |  |  |  |  |  |  |  |  |  |  |  |  | 2 | 0 | 2 |  |
| **Lae_06** | 340 | 346 |  |  |  |  |  |  |  |  |  |  |  |  |  |  |  |  |  |  | Total | Private | Shared |  |
| Mar | 0.5 | 0.5 |  |  |  |  |  |  |  |  |  |  |  |  |  |  |  |  |  |  | 2 | 0 | 2 |  |
| Gua | 0.5 | 0.5 |  |  |  |  |  |  |  |  |  |  |  |  |  |  |  |  |  |  | 2 | 0 | 2 |  |
| Jac | 1 | 0 |  |  |  |  |  |  |  |  |  |  |  |  |  |  |  |  |  |  | 1 | 0 | 1 |  |
| **Lae_08** | 330 | 334 | 338 | 342 | 346 | 350 | 354 | 366 | 370 | 374 | 378 | 386 | 394 | 402 | 406 | 414 | 418 | 422 | 426 |  | Total | Private | Shared |  |
| Mar | 0 | 0 | 0.05 | 0.14 | 0 | 0.14 | 0.27 | 0.09 | 0 | 0.09 | 0.05 | 0 | 0 | 0 | 0.09 | 0 | 0 | 0.05 | 0.05 |  | 10 | 7 | 3 |  |
| Gua | 0 | 0 | 0 | 0.35 | 0.05 | 0 | 0.5 | 0 | 0 | 0 | 0 | 0 | 0 | 0 | 0 | 0.05 | 0.05 | 0 | 0 |  | 5 | 2 | 3 |  |
| Jac | 0.03 | 0.13 | 0 | 0 | 0.19 | 0 | 0 | 0 | 0.03 | 0.03 | 0 | 0.25 | 0.03 | 0.31 | 0 | 0 | 0 | 0 | 0 |  | 8 | 6 | 2 |  |
| **Lae_10** | 453 | 463 | 465 |  |  |  |  |  |  |  |  |  |  |  |  |  |  |  |  |  | Total | Private | Shared |  |
| Mar | 0.69 | 0.13 | 0.19 |  |  |  |  |  |  |  |  |  |  |  |  |  |  |  |  |  | 3 | 1 | 2 |  |
| Gua | 0.8 | 0.2 | 0 |  |  |  |  |  |  |  |  |  |  |  |  |  |  |  |  |  | 2 | 0 | 2 |  |
| Jac | 0.55 | 0.45 | 0 |  |  |  |  |  |  |  |  |  |  |  |  |  |  |  |  |  | 2 | 0 | 2 |  |
| **Lae_11** | 232 | 240 | 242 |  |  |  |  |  |  |  |  |  |  |  |  |  |  |  |  |  | Total | Private | Shared |  |
| Mar | 0.67 | 0.29 | 0.04 |  |  |  |  |  |  |  |  |  |  |  |  |  |  |  |  |  | 3 | 1 | 2 |  |
| Gua | 0.5 | 0.5 | 0 |  |  |  |  |  |  |  |  |  |  |  |  |  |  |  |  |  | 2 | 0 | 2 |  |
| Jac | 0.91 | 0.09 | 0 |  |  |  |  |  |  |  |  |  |  |  |  |  |  |  |  |  | 2 | 0 | 2 |  |
| **Lae_14** | 457 | 469 | 475 | 478 | 481 | 484 |  |  |  |  |  |  |  |  |  |  |  |  |  |  | Total | Private | Shared |  |
| Mar | 0 | 0.04 | 0.42 | 0.54 | 0 | 0 |  |  |  |  |  |  |  |  |  |  |  |  |  |  | 3 | 0 | 3 |  |
| Gua | 0 | 0.13 | 0.5 | 0.38 | 0 | 0 |  |  |  |  |  |  |  |  |  |  |  |  |  |  | 3 | 0 | 3 |  |
| Jac | 0.16 | 0 | 0.09 | 0.63 | 0.09 | 0.03 |  |  |  |  |  |  |  |  |  |  |  |  |  |  | 5 | 3 | 2 |  |
| **Lae_15** | 406 | 412 | 430 | 433 | 436 | 439 | 442 | 445 | 448 | 457 | 463 | 466 |  |  |  |  |  |  |  |  | Total | Private | Shared |  |
| Mar | 0 | 0.22 | 0 | 0.06 | 0.39 | 0.06 | 0.11 | 0.17 | 0 | 0 | 0 | 0 |  |  |  |  |  |  |  |  | 6 | 1 | 5 |  |
| Gua | 0.13 | 0.19 | 0.06 | 0 | 0.5 | 0 | 0.06 | 0.06 | 0 | 0 | 0 | 0 |  |  |  |  |  |  |  |  | 6 | 1 | 5 |  |
| Jac | 0 | 0.2 | 0.27 | 0.07 | 0.07 | 0 | 0 | 0 | 0.13 | 0.2 | 0.03 | 0.03 |  |  |  |  |  |  |  |  | 8 | 4 | 4 |  |

**Table S3.** Proportion of pairwise kinship based on 10 microsatellite loci for *Laeonereis acuta*. U – Unrelated; HS – Half-sibling; FS – full-sibling; PO – Parental-offspring.

| Comparison between: | | U (%) | HS (%) | FS (%) | PO (%) |
| --- | --- | --- | --- | --- | --- |
| Maricá | Maricá | 63.6 | 19.7 | 10.6 | 6.1 |
| Guarapina | Guarapina | 42.4 | 24.2 | 27.3 | 6.1 |
| Jaconé | Jaconé | 61.8 | 29.4 | 7.4 | 1.5 |
| Maricá | Guarapina | 72.9 | 12.5 | 5.6 | 9.7 |
| Maricá | Jaconé | 98.4 | 1.6 | 0.0 | 0.0 |
| Guarapina | Jaconé | 97.9 | 1.6 | 0.5 | 0.0 |
